# Chemoprophylaxis of leprosy with rifampicin in contacts of multibacillary patients: study protocol for a randomized controlled trial

**DOI:** 10.1186/s13063-018-2623-6

**Published:** 2018-04-23

**Authors:** Daiane Santos dos Santos, Nádia Cristina Duppre, Euzenir Nunes Sarno, Roberta Olmo Pinheiro, Anna Maria Sales, José Augusto Da Costa Nery, Milton Ozório Moraes, Luiz Antônio Bastos Camacho

**Affiliations:** 1 Sérgio Arouca National School of Public Health, Oswaldo Cruz Foundation, Rio de Janeiro, Brazil; 2 Leprosy Laboratory, Oswaldo Cruz Institute, Oswaldo Cruz Foundation, Rio de Janeiro, Brazil

**Keywords:** Leprosy, Clinical trial, Chemoprophylaxis, Contacts, Rifampicin

## Abstract

**Background:**

The annual new-case detection rate for leprosy, while generally stable over the last decade, shows that transmission rates have remained stagnant despite the successful worldwide administration of multidrug therapy since the 1980s. As such, novel control strategies are urgently needed. Focusing on managing leprosy patient contacts, the most susceptible to contracting the disease, has been seen as a potential strategy in limiting the spread of leprosy as shown by a number of recent epidemiological studies. Immunoprophylaxis with Bacillus Calmette-Guérin (BCG) has been seen as an effective preventive measure due to its ability to stimulate the development of cellular immunity which is essential in controlling the disease, especially in its multibacillary (MB) forms. The association of immunoprophylaxis with chemoprophylaxis in a single dose of rifampicin has been shown to be a promising preventive strategy, although a variety of studies have found instances of early case detection just a few months after BCG vaccination.

**Methods/design:**

The present study is a phase IV chemoprophylactic clinical trial consisting of administration of a single dose of rifampicin in MB leprosy patient contacts under care at the Souza Araújo Outpatient Clinic/FIOCRUZ as part of a randomized (2:1), double-blind, placebo-controlled study. It is comprised of two groups: 1) rifampicin + BCG; and 2) placebo + BCG.

**Discussion:**

The aim is to evaluate whether the use of chemoprophylaxis with a single dose of rifampicin in MB leprosy patient contacts prior to the BCG vaccine would be able to prevent the onset of leprosy in those cases that may occur just a few months after vaccination. Contact subclinical infections (polymerase chain reaction) and the immunological parameters (anti-PGL-1, anti-LID-1, and IFN-γ) will be evaluated and the results will be compared after 12 months of follow-up.

**Trial registration:**

The Brazilian Registry of Clinical Trials (ReBEC), RBR-69QK5P. Retrospectively registered on 1 June 2017.

**Electronic supplementary material:**

The online version of this article (10.1186/s13063-018-2623-6) contains supplementary material, which is available to authorized users.

## Background

Following the introduction of multidrug therapy (MDT) in the 1980s, the global prevalence of leprosy drastically declined from more than 5 million cases worldwide to a little more than 200,000 in 2014. Likewise, over the last 10 years or so, the number of new global cases has markedly decreased from 299,036 in 2005 to 210,758 in 2015 [1, 2]. According to Lockwood and coworkers [3], the near-term goal regarding the elimination of leprosy needs to be revised primarily since India and Brazil continue showing high detection rates notwithstanding major advances in detection strategies, more easily accessible treatment, and constant innovation. The goal must above all be realistically achievable and based on proven scientific evidence and constant updating as new information emerges.

Brazil ranks second worldwide in the total number of leprosy cases, with India being the first. Brazil, however, takes first place in the Americas, where it was responsible for a full 92% of all new patients in 2015 [2]. According to Brazilian Ministry of Health (MH) data, 28,761 new leprosy patients were detected (14.7/100,000 inhabitants) in 2015. However, state-wide detection rates varied widely from 1.08/100,000 inhabitants in the State of Rio Grande do Sul to as high as 93.0/100,000 inhabitants in Mato Grosso State [4].

The pillars that sustain disease control measures are the administration of MDT to all newly detected leprosy patients along with close surveillance of their contacts. The prime objectives are early detection and timely treatment. Since 1991, the MH has recommended that the Bacillus Calmette-Guérin (BCG) vaccine be administered to all intradomiciliary contacts within 5 years of their index (primary) case diagnosis.

A study conducted in Brazil by Duppre and coworkers [5] showed that, despite the protection conferred by the BCG vaccine, a significant number of cases have occurred within a short period of time afterwards, mostly among contacts of multibacillary (MB) patients. Another study by Duppre et al. [6], using anti-PGL-I serology to assess the risk of becoming ill, found an excess of new leprosy cases within the first few months after vaccination, mainly among those positive for anti-PGL-1, indicating the presence of prior subclinical infection.

In 1989, Bagshawe et al. [7] reported that previous immunity to mycobacterial antigens was primarily responsible for the clinical manifestations of paucibacillary (PB) leprosy and that the nonspecific immune stimulation induced by BCG vaccination might precipitate clinical manifestations of leprosy in individuals with subclinical infection.

Another proposed measure of control is chemoprophylaxis among leprosy contacts via a single dose of rifampicin. The COLEP, a randomized, double-blind, placebo-controlled trial conducted in Bangladesh, observed a 57% risk reduction during the first 2 years of follow-up in conjunction with a higher protective chemoprophylactic effect among contacts of nonconsanguineous social contacts of MB index cases. Moreover, childhood BCG had a complementary effect on rifampicin, thereby reducing the number of contacts that became ill after vaccination [8]. These findings clearly indicate the need to evaluate the combined effect of BCG immunoprophylaxis and rifampicin chemoprophylaxis in contacts of new index cases.

In this same vein, a study by Richardus et al. [9] evaluated the protective role of BCG at the initial contact examination and 8 weeks later, in association with a single dose of rifampicin. Among the 5196 contacts included in the above study, 21 became ill (0.40%) 12 weeks after BCG, 12 of whom (57%) had no previous BCG scar, while 18 (87%) developed tuberculoid leprosy. These results are in line with those published by Duppre and coworkers [6].

In the area of serodiagnostics, the detection of IgM antibodies against phenolic glycolipid (PGL-1) represents the most reliable and highly evaluated standardized leprosy test available to date [10]. Nevertheless, leprosy IDRI (Infection Disease Research Institute) diagnostic-1 (LID-1), a novel protein (*Mycobacterium leprae,* ML0405, and ML2331), is considered to have diagnostic potential [11]. In parallel, polymerase chain reaction (PCR) has demonstrated greater sensitivity than bacilloscopy [12, 13]. It may be that the PGL-1, LID-1, and PCR combination is able to identify the most susceptible leprosy contacts.

Nonetheless, the immunoregulatory mechanisms involved in the early stages of the disease that could be targeted for the development of new tests have not yet been established. Moreover, the understanding of the impact of chemoprophylaxis on contacts is also limited due to the insufficiently described immunological profile of those who have been administered chemoprophylaxis, whether associated or not with immunoprophylaxis.

Besides, it is worth noting that the lack of a cell-mediated immune response to *M. leprae* antigens in individuals exposed to the infectious agent may be predictive of susceptibility. The production of interferon (IFN)-γ in peripheral blood mononuclear cells has been used as a measure of cellular responsiveness. A previous study by our group showed that the five contacts who developed leprosy during follow-up belonged to the group that was negative to or had reduced levels of IFN in response to mycobacterial antigens [14].

We report an ongoing study set out to assess the effectiveness of leprosy chemoprophylaxis with rifampicin among contacts of MB patients. The study will also assess the effect of rifampicin on laboratory parameters, namely anti-PGL-I, anti-LID-I, IFN-γ, and PCR, thought to indicate susceptibility to leprosy.

## Methods

### Study design

This is a phase IV, randomized (2:1), double-blind clinical trial involving either a single dose of rifampicin or placebo for the chemoprophylaxis of leprosy. Immunoprophylaxis with BCG as recommended by the Brazilian Leprosy Control Program was conducted in both comparison groups.

### Study site

The clinical trial will be carried out at the Ambulatory Souza Araújo (ASA) Outpatient Clinic associated with the Leprosy Laboratory of the Oswaldo Cruz Foundation. In partnership with the Leprosy Laboratory, the ASA is known for its clinical, laboratory, educational, and research activities in the areas of immunology, pathology, molecular biology, microbiology, and clinical practice, in addition to providing ready access to basic care and complex diagnostic tests. As a leprosy reference center, the ASA can optimize the performance of chemoprophylaxis and guide its future use in other health care units.

### Study population

Study participants are recruited from index case contacts already registered at the ASA who either live in the city of Rio de Janeiro or its metropolitan area. The clientele is mostly comprised of referrals from local health care services (both public and private) and those who arrive spontaneously.

After a leprosy diagnosis (clinical and/or bacteriological and/or histopathological), patients are informed about their clinical form, treatment regimen, mode of transmission, preventive measures, and the importance of presenting their contacts for clinical evaluation. All referrals made by the patient, whether consanguineous or not, household contacts, or those having close familial and/or social ties, are scheduled for a dermatoneurological examination as soon as possible after their index case diagnosis.

### Inclusion and exclusion criteria

The eligibility criteria are sufficiently broad to encompass most potential beneficiaries and yet protect the study from any selective or informational bias. The inclusion criteria are: 1) contacts of MB leprosy patients who agree to undergo chemoprophylaxis; 2) ages 6 months to 70 years; 3) an expressed willingness to undergo both clinical and anti-PGL-1 evaluations; 4) availability for follow-up; and 5) a firm commitment to return for vaccination within 2 months and be clinically evaluated at the end of 12 months. The minimum period for detecting the postulated effect will be 12 months.

Exclusion criteria are: 1) clinical or laboratory confirmation of leprosy at baseline; 2) BCG vaccination within the preceding 12 months (to prevent vaccine-related changes in the immune response), except for infants between 6 and 12 months of age (leprosy detection in infants indicates active transmission and the possibility of having other MB cases in the contact cluster); 3) contacts with immunosuppression and/or a history of tuberculosis in any of its forms; 4) pregnancy at any stage or refusal to undergo a urine pregnancy test; and 5) refusal to sign the informed consent form (ICF).

### Randomization and blinding

The allocation of the participants into groups is based on random sequence generation via computer software (WINPEPI program, version 11.18). The ratio is 2:1 with two intervention groups and one control group for the purpose of expanding the pool of information generated by chemoprophylaxis. Participants will be randomly assigned to intervention or placebo in permuted blocks of variable sizes defined by the statistician, concealed from team members. Blocked randomization guards against any imbalance in the number of participants assigned to each group, a major concern for interim analysis, and also protects against the temporal variations in individual characteristics as well as in study procedures that may arise in long enrollment phases.

The intervention code (rifampicin/placebo) for each patient is placed in sealed opaque envelopes and numbered sequentially (natural numbers). The envelopes are only opened after each contact has voluntarily signed the ICF to avoid foreknowledge of the allocated group. The number printed outside the envelope identifies the participant and the opened envelopes are saved to ensure traceability. Self-adhesive tags identifying the code of each volunteer are pasted onto the recruitment form, ICF, blood collection tubes, and each patient identification card. Group assignment will be concealed from study participants and from the researchers, physicians, and research assistants performing the study procedures (double-blind) as a way of protecting it from any unconscious biases or expectations as to the outcome. Two nonblinded team members that do not participate in data collection and analyses are responsible for dispensing rifampicin and the placebo, opening and storing envelopes for randomization, labeling study documents, and administrating the medications. The allocation ratio (2:1) will make the intervention group twice the size of the control group, thus hampering the blinding of the statistical analysis of the data.

Besides preventing informational bias, blinding may additionally avert adherence problems in the placebo group. During follow-up, unblinding will be allowed whenever the clinical management of adverse events requires disclosure of the individual’s group to the attending physician and to the independent monitoring committee.

### Intervention

All contacts included in the study receive a single supervised dose of rifampicin or placebo. Either rifampicin or placebo is administered during the initial visit, but only after each contact has signed the ICF, undergone anamnesis, been clinically examined, and had their data collected.

The rifampicin dose consists of 600 mg, or 10 mg/kg body weight in contacts weighing less than 40 kg. Infants and children receive the dosage in a solution determined by weight [15]. All contacts must receive the BCG vaccine a maximum of 2 months after the single dose of rifampicin or placebo.

Maintaining the blinding poses some challenges because rifampicin often leads to reddish-colored urine, tears, and sweat. Rifampicin and placebo capsules have identical organoleptic properties even though starch placebos do not mimic the reddish coloration in the urine and elsewhere.

### Outcomes

The primary outcome of the present study is the development of new leprosy cases during follow-up.

The assessment of clinical disease is based on the identification of cutaneous lesions, with observed changes in sensitivity and thickened nerves [16]. Clinical diagnoses are ascertained by professionals with leprosy expertise and include a dermatologist and a nurse responsible for the dermatological, clinical, and previous history evaluations, as well as a physiotherapist in charge of neurological assessment.

When the contacts return for evaluation, those with suspected clinical leprosy will be submitted to bacteriological and histopathological testing and then classified according to the Ridley and Jopling scale (1966) [17] as: borderline-borderline; borderline-lepromatous; lepromatous-lepromatous; tuberculoid; borderline-tuberculoid tuberculoid; or undetermined. Confirmed cases will also be grouped according to their grade of disability and bacilloscopic index (BI) as MB if BI-positive, or PB if BI-negative.

Secondary outcomes are: 1) the serological status of anti-PGL-1 prior and subsequent to chemoprophylaxis and before immunoprophylaxis; 2) the serological status of anti-LID-1 prior and subsequent to chemoprophylaxis and before immunoprophylaxis; 3) the PCR positivity, which is considered a marker of increased risk for developing leprosy; 4) IFN-γ production in response to *M. leprae* before and after chemoprophylaxis; 5) adverse events potentially associated with rifampicin; 6) intercurrent clinical conditions due to intervention; and 7) concordance between rapid test and serology for anti-PGL-1.

### Follow-up

Recruitment strategies for the study include adapted routine ASA procedures that are recorded on the appropriate forms. Initially, identification, demographic, and socioeconomic variables are recorded along with type and length of time of index case cohabitation. The contacts are duly informed concerning the clinical forms of the disease, incubation periods, signs and symptoms, modes of transmission, and treatment regimens.

The contacts are submitted to a detailed physical examination including verification of a BCG scar, sensitivity testing, and a traditional dermatological examination to identify suspected leprosy lesions. The neurological evaluation covers inspection and palpation/percussion, together with the functional evaluation of the peripheral nerves and identification of deformities [18]. The cases detected in the initial evaluation are considered coprevalent contacts of their index cases, thus becoming index cases themselves and triggering a contact search.

After this first stage, signed informed consent is obtained from all participants aged 18 and over in addition to the parents/guardians of those up to 17 years of age. A signed assent form is also required for the 12- to 17-year-old participants.

The informed consent form includes: a medical description of leprosy; the role played by contact surveillance in disease control; the study objectives; the risks and benefits related to intervention; the commitment to participate in the study, including an initial evaluation, another after 2 months, and a third upon completing 1 year; concealed randomization of group participation; the possibility of the occurrence of adverse events; and the procedures to be followed if further assistance is needed accompanied by the data necessary to contact team members.

After signing the forms, 16 ml of blood is collected from adults and contacts aged 12 to 17 years for the baseline identification of the following immunological markers: anti-PGL-1, anti-LID-1, and IFN-γ during the first evaluation, at the end of the second month (prior to BCG immunization), and 12 months subsequent to the initial intervention. All participants will undergo digital pulp blood sampling for rapid anti-PGL-1 testing during the initial evaluation and after 12 months following intervention. Children under 12 years of age will have only a digital pulp blood sample collected for rapid anti-PGL-1 test. This test has been validated by Bührer-Sékula et al. [10], and a simple verification of the agreement with the results of the enzyme-linked immunosorbent assay (ELISA) (anti-PGL-I) in adults will be conducted in this study to inform of the limitations of the results in children, for whom ELISA (anti-PGL-I) will not be available.

Lymph from the right earlobe is obtained by dermal scraping for DNA/ML molecular investigation (PCR) during the first evaluation, but only from the participants over 12 years of age, since children under 12 years of age are less able to bear the discomfort. Another lymph sample is collected at the end of the second month in the event the result of the first examination is either positive or inconclusive. Lymph collection follows MH guidelines, according to which the specimen is stored in a sterile bottle immersed in a 70% alcohol solution [19].

**Fig. 1:**
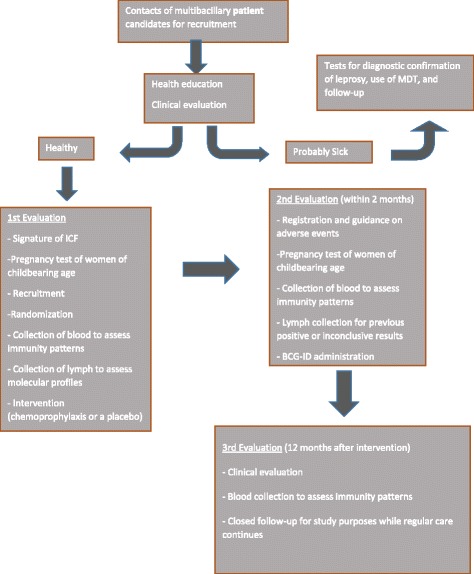
Flowchart of the clinical trial of chemoprophylaxis with rifampicin in contacts of multibacillary leprosy patients. BCG-ID, Bacillus Calmette-Guérin intradermally; ICF, informed consent form; MDT, multidrug therapy

The BCG vaccine is administered 2 months after the first clinical assessment has been made in contrast to the MH recommendation in that regard [18]. This alteration makes it possible to evaluate the immunological parameters of each participant in response to rifampicin alone on the one hand, and the placebo alone on the other. Since rifampicin has a significant bactericidal effect, a 2-month interval between the administration of rifampicin and BCG is planned to avert interference of the antibiotic in the immune response to this attenuated live vaccine. Because of the slow progression of leprosy disease, however, the postponement of BCG vaccination for up to 2 months after receiving rifampicin should not substantially interfere with the protection conferred by BCG. Contacts under 1 year of age will be administered BCG only if they have not been vaccinated before. We clarified that participants are encouraged to seek care at the ASA Outpatient Clinic any time they present signs or symptoms indicated in the baseline interview. The flowchart of the trial is show in Fig. 1.

The study is expected to last a total of 6 years with the understanding that all contacts participate in a minimum 1-year follow-up period. To minimize losses during that time, a search will be carried out in the official MH leprosy database, or SINAN, to identify new leprosy cases among ASA contacts that were originally diagnosed in another health care unit. The search variables will include the full names of the contact and his/her mother in addition to the date of birth of the former. Those who were not treated at ASA and are not on the SINAN database will be considered healthy.

**Fig. 2:**
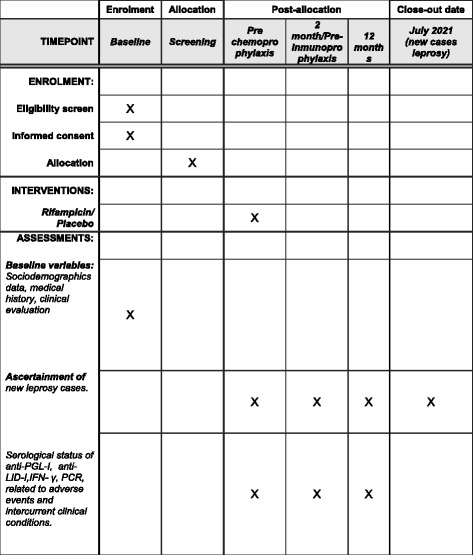
Schedule, enrolment, intervention and assessments for the clinical trial of chemoprophylaxis with rifampicin in contacts of multibacillary leprosy patients. IFN, interferon; LID, leprosy Infection Disease Research Institute diagnostic; PCR, polymerase chain reaction; PGL, glycolipid phenolic

The schedule of enrollment, interventions, and assessments of the study is shown in Fig. 2, Additional file 1. The “Close-out” is the end of follow-up for the study since participants will continue to have access to the outpatient clinic on a routine basis, as will all contacts. All participants are assessed at fixed points: upon recruitment/allocation, and after 2 and 12 months. Afterwards, case ascertainment continues until July 2021 as participants are encouraged to return whenever signs/symptoms (to which they are alerted) appear, or SINAN database discloses a new case that belongs to the study cohort.

### Adverse events

Adverse events and clinical complications will be monitored throughout the study period. The symptoms and signs temporally related to the intervention are recorded on a specific form, regardless of any presumed causal association with the intervention.

While the probability of the occurrence of adverse events related to the administration of a single dose of rifampicin is low, the most common ones have been loss of appetite, nausea, vomiting, diarrhea, and orange or reddish coloration of urine, saliva, feces, sweat, and tears. There may also be occurrences of colitis, facial flushing, hives, rashes, jaundice, liver failure, red spots on the skin, bleeding from the nose, gums, or vagina, anemia, flu symptoms, fever, weakness, headache, tremors, mental confusion, motor coordination disorders, transient visual changes, peripheral neuritis, and venous thrombosis [15].

Following blood collection, pain and bruising may take place at the puncture site, and auricular lobe lymph collection may also cause transient pain at the collection site.

In accordance with MH guidelines, BCG vaccination may lead to adverse reactions at the application site and in satellite lymph nodes, while severe systemic reactions [18] have very rarely been known to occur.

### Laboratory methods

#### Serology for anti-PGL-1 and anti-LID-1

Serology is performed prior to administration of rifampicin/placebo, and 2 and 12 months after chemoprophylaxis. As such, 96-well plates are coated with 1 μg/ml recombinant LID-1 or PGL-1 protein in a bicarbonate buffer for 18 h at 40 °C and blocked for 1 h at room temperature with phosphate-buffered saline (PBS)-Tween containing 1% bovine serum albumin (BSA). Serum samples are diluted in 0.1% BSA, added to each well, and incubated at room temperature for 2 h accompanied by shaking. Plates are washed and IgM HRP (Rockland Immunochemicals, Gilbertsville, PA, USA), is diluted in 0.1% BSA and then added to each well before incubation at room temperature for 1 h with shaking. After washing, the plates are developed with the peroxidase substrate, and the reaction is terminated by adding 1NH_2_SO_4_.

#### ML flow test

A digital pulp blood sample will be submitted to the rapid test ML Flow implemented as part of the contact’s routine examination in the ASA Outpatient Clinic in 2003. The test is read after 5 and 10 min and the result is considered valid when the control line is visible, and considered positive when a distinct staining reddish line is observed. When no staining or only a faint staining is visible the result is considered negative, as described by Bührer-Sékula and coworkers [10].

#### Whole blood test for evaluation of the cellular immune response

Heparinized whole blood is diluted 10 times in serum-free culture medium (RPMI 1640 supplemented with 100 U/ml penicillin, 10 μg/ml streptomycin, and 2 mM l-glutamine). The culture is stimulated or not with 10 μg/ml irradiated *M. leprae*. After 5 days, the plasma is collected to assess the concentration of IFN-γ using ELISA.

#### PCR for DNA detection of *M. leprae*

The biological samples are collected according to MH and World Health Organization (WHO) recommendations and stored in a freezer at −70 °C or liquid nitrogen until processed. Samples of contact dermal scrapings of the right earlobes are likewise collected as per MH recommendations regarding leprosy diagnosis [16].

DNA extraction from the dermal scrape samples is performed using the DNeasy Blood and Tissue Kit as per the manufacturer’s instructions (QIAGEN®) and aided by an extractor robot.

Real-time PCR assay is standardized to amplify a 16S gene sequence to determine the relative number of bacteria in the contact biological samples [13]. PCR reactions are prepared with Applied Biosystems reagents (Mastermix®) to a final volume of 25 μl.

### Statistical analysis

The evaluation of the efficacy of chemoprophylaxis will be based on the occurrence of new cases of leprosy in both groups and will be measured at least at 1 year after the single dose of rifampicin. Over the period of 1 year, three evaluations will be carried out: at baseline, 2 months, and 12 months.

The total number of randomized participants will be analyzed to preserve the safeguards against selection bias and confounding (“intention-to-treat analysis”). An alternative analytical strategy (“per-protocol analysis”) will disregard the data obtained from participants that did not meet the eligibility criteria and did not adhere to either of the intervention regimens or study procedures.

Evaluating the group that adhered to the protocol prevents classification bias that may result from analyzing the participants in the originally allocated group despite their not having followed the specifically planned procedures**.** Any discrepancies in the results of the two analytical strategies will be interpreted in light of the distribution of the baseline characteristics of the participants adhering to the protocol. The balanced distribution of covariates across comparison groups achieved by randomization could be lost as a result of violations in protocol.

The main explanatory variable defined by the study design is the intervention (rifampicin/placebo). Whereas univariate analyses will be performed to describe their baseline characteristics, bivariate analyses will explore the association between intervention and outcomes (response variables). Multivariate analyses (Cox regression) will be conducted to adjust the association estimates (hazard ratio for new cases) for covariates with any imbalance in group distribution. Subgroup analyses of effectiveness (derivatives of the measures of association) according to age, along with the BI and type of cohabitation with the index case, will be carried out.

Cox regression is used to estimate the conditional hazard ratio and the survival function based on longitudinal data from a dynamic cohort of individuals under surveillance for different periods. The hazard ratio estimates the magnitude of the association between chemoprophylaxis and leprosy. The measurement takes into account the different time periods that have elapsed between exposure (treatment/placebo) and the outcome (illness) for each participant until disease develops or follow-up is discontinued [20]. The survival curve will be required to handle open and continuous recruitment as well as censorship, and the survival rates can be estimated by conditional probability according to the strategy proposed by Kaplan and Meier. The statistical significance will be assessed by the Log-Rank test [21].

For all the analyses, a 5% significance level will be adopted, and 95% confidence intervals constructed for the estimates.

An interim analysis will be conducted at the end of the third year of the study to evaluate individuals who have had at least 1 year of follow-up. This analysis will verify whether the cessation of participant recruitment is warranted by the data collected thus far and will be an opportunity to revise sample calculations according to the results. Interim analysis will seek evidence of the effectiveness, or lack thereof, of chemoprophylaxis or any clear indications that the postulated effect is so unlikely that recruitment is no longer justified. The assessment will be based on the number of events accumulated thus far and the trend in the difference between comparison groups, and the discontinuation of the trial will be submitted to the Data Monitoring Committee.

The critical level of a *z* score to reject the null hypothesis in the interim analysis will be adjusted to 2.782 (*p* = 0.005) in line with the O’Brien-Fleming protocol [22]. The conservative criterion in the interim analysis protects the study from the often-misleading results obtained from smaller samples.

### Analyses of outcomes based on immunological and molecular parameters

The quantitative variables analyzed will be those related to the ELISA results on anti-PGL1, LID-1, and IFN-γ. The optical density (OD) of each will be read at 450 nm. PGL-1 samples with OD > 0.25 will be considered positive while LID-1 samples will be considered positive with OD > 0.3. As for IFN-γ, the reading will be performed according to the manufacturer’s recommendations (eBioscience®, San Diego, CA, USA).

Frequency distribution will be constructed to detect asymmetries and the need for scale transformation. The primary analysis will address measures of the central tendencies (mean and median), and those of dispersion (i.e., interquartile range, variance, and standard deviation).

For the continuous variables, paired measurements for each individual will result from blood samples obtained before receiving rifampicin and during the contact visit 2 months after receiving rifampicin and before receiving the BCG vaccine. The results obtained 12 months after the first blood collection will also be submitted to paired analysis with the *t* test comparing the mean values of any changes between the two groups.

The main explanatory variable is intervention (rifampicin/placebo). Bivariate and multivariate analyses (linear regression model) will consider the following major covariates: age group, bacilloscopic index (BI), and type of coexistence with the index case. The response variables will include the anti-LID-1, anti-PGL-1, and IFN-γ measurements on a continuous scale. The effect of the intervention will be evaluated and adjusted for the effects of the other explanatory variables.

Regarding the PCR results (positive/negative), bivariate and multiple analyses (logistic regression model) will include the same previously mentioned explanatory variables: age group, BI, and type of cohabitation with the index case. A 5% significance level will be adopted, and 95% confidence intervals will be constructed for the estimates. Data management will use Microsoft Access®, and statistical analyses will be conducted using SPSS version 22.0 (IBM Corp., 2013).

### Calculation of sample size

The sample size of this study was calculated on the basis of the primary outcome of the leprosy chemoprophylaxis among contacts using rifampicin associated with BCG vaccine. For the occurrence of this outcome a minimum follow-up time was set at 1 year.

Sample size calculations were based on the proportion of contacts that would likely develop leprosy per intervention group, currently estimated at 5% for the placebo group and 2% for the rifampicin group. With an allocation ratio of 2:1, the difference of 3 percentage points, a statistical power of 80%, and a significance level of 5%, (corrected to 0.025 to account for the interim analysis), the study requires 829 participants in the intervention group and 415 controls for a total 1244 participants. Estimating a 20% loss during follow-up (based on previous ASA data), the total sample size would total 1493 participants (995 in the treatment group and 498 controls). This sample size is appropriate for the analysis of time to failure enabling the study to detect more subtle effects (hazard ratio 0.67). Assuming a 30% seropositivity for PGL-1 among controls, this sample size will provide 85% power to detect an 8 percentage point difference from the treatment group. For 2.5% LID-positive controls, the study has 77% power to detect a 2 percentage point difference. For 35% IFN-γ-based susceptibility the study has an 80% power to detect an 8 percentage point difference. The calculations were performed via PASS11 software [23].

## Discussion

The proposed study seeks to generate clinical and laboratory evidence that chemoprophylaxis combined with immunoprophylaxis may alter the natural course of subclinical infection by *M. leprae* by reducing the incidence of new cases and altering the laboratory parameters of infection. It is hoped that the results of the present study broaden the theoretical basis of the leprosy control program in Brazil.

The MH started a pilot project, referred to as PEP-hans, in a number of defined areas in the states of Pernambuco, Mato Grosso, and Tocantins in June 2015 [24]. The objective was to evaluate as closely as possible the effectiveness of postexposure chemoprophylaxis with one dose of rifampicin. The intervention is expected to reduce the risk of leprosy among contacts who present themselves to any health care facility nationwide under the Unified Health System (UHS), while keeping in mind the operational aspects involved in that initiative. Although PEP-hans evaluates the effectiveness of single-dose rifampicin in a population similar to the one in the present study, it differs in several methodological aspects such as the administration of rifampicin to contacts of index cases diagnosed up to 12 months beforehand and who received the BCG vaccine at baseline.

The authors consider that one of the advantages of the present study over PEP-hans is the evaluation of the immunological parameters before and after the administration of rifampicin (again, prior to the BCG vaccine) in the presence or absence of *M. leprae*. This strategy may expand our knowledge of the immune mechanisms that identify the contacts most susceptible to illness and may also contribute to the future development of diagnostic kits.

The ongoing MALTALEP clinical trial developed by Richardus et al. [25] also aims to evaluate the combined effect of single-dose rifampicin chemoprophylaxis and BCG immunoprophylaxis in new leprosy contacts. The present study shares the hypothesis that there is a combined effect of chemoprophylaxis and immunoprophylaxis but objectifies the gathering of information on the immunological parameters that have been influenced by the separate interventions in the same individual.

The above study and ours will carry out immunological and genetic analyses but at different times. MALTALEP will collect blood from 150 contacts in each randomized group within an 8-week period and within 1 and 2 years of follow-up. In our trial, however, blood collection will take place upon recruitment and then 2 and 12 months later. The results of these studies may be complementary to each other and, if so, be able to identify more effective strategies to manage contacts at greater risk and elaborate secondary preventive measures with rifampicin.

Research settings, particularly for experiments, may distance themselves from the “real world”, so that results may not be reproducible under typical operational conditions prevailing in average public health care units. In this regard, the proposed intervention is simple enough to be easily incorporated into routine practice. Conversely, the laboratory tests may pose additional difficulties for the public health care services. The present study addresses day-to-day medical issues, but the limitations of the external validity of the results should be acknowledged.

Leprosy is a millennial disease and, although it shows a low mortality rate in recent times, it continues to present high morbidity with the loss of the capacity to work accompanied by pernicious social stigmatization, often making it difficult to approach possible leprosy patient contacts.

Chemoprophylaxis represents an opportunity to increase the impact on contact control to prevent the rise of new cases while infection markers will allow the contacts with the highest potential to benefit from this intervention. The results of this trial could make a considerable contribution to the prevention and containment of leprosy and to a more robust clinical management of contacts who become infected**.** With the additional resources available for clinical investigation and prophylaxis, it may be possible to more readily engage leprosy contacts to fully comply with all medical recommendations.

## Trial status

The recruitment started in July 2015; participants are currently being recruited, and we expect to close in July 2020. An interim analysis will be performed in July 2018, and the final analysis will be performed in July 2021. This is protocol version 4.

## Additional file

Additional file 1: SPIRIT 2013 Checklist: Recommended items to address in a clinical trial protocol and related documents. (PDF 172 kb)

## Abbreviations

ASA: Ambulatory Sousa Araújo
BCG: Bacillus Calmette-Guérin
BI: Bacilloscopic index
BSA: Bovine serum albumin
ELISA: Enzyme-linked immunosorbent assay
ICF: Informed consent form
IDRI: Infection Disease Research Institute
IFN: Interferon
LID: Leprosy IDRI diagnostic
MB: Multibacillary
MDT: Multidrug therapy
MH: Ministry of Health
OD: Optical density
PB: Paucibacillary
PCR: Polymerase chain reaction
PGL-I: Glycolipid phenolic I
WHO: World Health Organization
